# Mpox virus: a growing threat to fragile healthcare systems in Pakistan

**DOI:** 10.1097/MS9.0000000000001378

**Published:** 2023-10-02

**Authors:** Syed Hassan Ahmed, Mariam Shahabi, Hurais Malik, Shiza Abid, Md. Al Hasibuzzaman

**Affiliations:** a Dow University of Health Sciences; b Fazaia Ruth Pfau Medical College, Karachi; c Ayub Medical College, Abbottabad, Pakistan; d Institute of Nutrition and Food Science, University of Dhaka, Dhaka, Bangladesh; e The First Affiliated Hospital of Ningbo University, Ningbo, People’s Republic of China

The enveloped double-stranded DNA virus known as mpox, formerly referred to as monkeypox, belongs to the Orthopoxvirus genus and has garnered heightened attention due to recent outbreaks beyond Africa, underscoring its potential as a worldwide health concern^1^. Globally, 88 549 confirmed cases of mpox, including 152 fatalities, have been recorded across 111 countries as of 19 July 2023, yielding a case fatality ratio (CFR) of ~0.172%. This has prompted the implementation of extensive public health interventions^2^. During the recent outbreak, a mortality rate of 1.2 for every 1000 cases has been observed, with immunocompromised patients, such as those with advanced human immunodeficiency virus (HIV) infection, at a higher risk^3,4^.

Transmission of the virus occurs through direct contact with both animals and humans. Human-to-human transmission involves various modes, such as respiratory droplets, skin-to-skin contact, sexual encounters, and vertical transmission from infected pregnant individuals to their fetuses^5^. The outbreak, primarily attributable to intimate encounters, especially among homosexual males, has seen a median age of 34 years and a male predisposition. Notably, 98% of infections have occurred within the gay or bisexual population, with 41% being co-infected with HIV^6^.

While the first case of mpox was identified in the 1970s, the World Health Organization (WHO) declared it a public health emergency of international concern (PHEIC) on 24th July 2022. Although it is no longer considered a PHEIC, there remains a lingering risk of new outbreaks of both mpox and coronavirus disease 2019 (COVID-19), as described by Tedros Adhanom, the Director-General of the WHO^7^. Africa has experienced a 7.4% increase in cases, with the Democratic Republic of the Congo witnessing a sharp rise of 23.6% in recent weeks. Such patterns emphasize the persistent threat posed by the disease. Addressing the disease’s shifting epidemiology is paramount, as ignoring it can lead to global repercussions, as exemplified by the current state of mpox^8^. It is imperative to recognize that the present ability to control a disease does not automatically assure its long-term manageability. African epidemiologists have consistently alerted to shifting disease patterns, although their cautions have frequently been disregarded, leading to the global prominence of mpox as a formidable issue. Significantly, recent infections have exhibited heightened infectivity, potentially attributed to genetic modifications. This escalated infectivity might expedite broader transmission, mandating thorough surveillance and expedient response strategies^1^.

The virus employs a sophisticated mechanism, infiltrating host cells, exploiting cellular machinery, and proliferating extensively. This leads to the production of viral progeny that disseminate through the bloodstream to various organs, including the skin, respiratory tract, and lymph nodes, underpinning the systemic infection. The host’s immune response involves innate immune cell detection, cytokine release, and immune cell recruitment to the infection site^9^. This interplay shapes clinical manifestations, with immune cell infiltration contributing to characteristic skin lesions. The disease presentation initiates with an initial prodromal phase typified by fever, cephalalgia, myalgia, and lymphadenopathy, succeeded by the emergence of multiple pseudo-papular^10^, ulcerative, and vesiculopustular cutaneous lesions, which evolve from macules to papules, vesicles, pustules, crusts, and culminate in scab formation^6^. The majority of individuals afflicted with mpox undergo a self-limited disease trajectory, and symptoms typically abate within a span of 2–4 weeks^1^. Fatality rates attributed to mpox infection exhibit variability, ranging from 1 to 10%^6^.

As a developing country with a limited budget for health expenditures, Pakistan faces a plethora of healthcare challenges compounded by the poverty and illiteracy of its burgeoning population. The COVID-19 pandemic stretched Pakistan’s healthcare sector to its limit, leading to inadequate personal protective equipment supplies, ventilator unavailability, and overburdened hospitals. Furthermore, the government faced numerous issues in enforcing lockdowns to contain the spread of the disease^11^. A second healthcare challenge came in the form of the disastrous floods last year, which not only exacerbated the disease burden including diarrhea, typhoid, cholera, dengue, and malaria but also resulted in a significant reproductive health crisis^12^. It, therefore, stands to reason that any arising outbreaks, such as mpox, should warrant concern, given the existing burden on the health system. To date, five cases have been reported in Pakistan, with the first case reported in April 2023 and all reported cases being diagnosed in travelers returning from Saudi Arabia^13^. Nevertheless, vigilance and adequate preparation are still required in Pakistan. Despite the low death rate estimated by WHO, a recent study has found that the mortality rate of mpox in HIV patients may be as high as 15%^14^. This is concerning, given the rising number of HIV cases in Pakistan, especially in children^15^. It is also worth mentioning that 89 000 people affected by the floods of 2022, have still not been rehabilitated, and are residing in shelter camps^12^. The risk of a mpox outbreak in such a setting, coupled with a lack of access to good hygiene and sanitation, is a public health crisis in the making. Even in large urban centers such as Karachi, residents of densely populated slums and low-income neighborhoods face similar issues of poor sanitation and, hence, are vulnerable to similar problems.

To date, the health authorities have confirmed no evidence of local transmission in Pakistan. All the cases had a history of international travel; therefore, the airports must remain on high alert, and all symptomatic passengers must be carefully screened. In the case of any diagnosed case, strict quarantine and rigid contact tracing must be ensured to contain the spread among the local population. Currently, there are no approved guidelines specifically designed for treating mpox, and preventive measures remain the mainstay of management. However, the antiviral drug tecovirimat (TPOXX) is utilized to treat severely ill patients who have immunosuppressive conditions like uncontrolled human immunodeficiency virus (HIV), leukemia, or conditions such as lesions, atopic dermatitis, psoriasis, herpes, etc. This drug may also be considered for use in children and pregnant or breastfeeding women due to its effectiveness and safety profile. Individuals with milder symptoms may be prescribed over-the-counter medications like ibuprofen and acetaminophen^16^. However, given the controlled number of cases reported to date and the mild severity observed, the adherence to guidelines has not yet been put to the test. When caring for individuals with mpox, healthcare professionals should ensure their safety by using appropriate personal protective equipment (PPE) such as gloves and masks. They must maintain strict hand hygiene practices and isolate patients in well-ventilated rooms, following precautions for both direct contact and respiratory droplets. Minimizing exposure is key, achieved through restricted personnel access, controlled visitor interactions, and proper disposal of infectious waste^4^. Meanwhile, conducting training sessions for doctors and paramedic staff regarding mpox management and adapting to the newer diagnostic methods with better accuracy, as seen in the case of the GeneXpert MPOX/OPX test, can help with the timely detection of cases^17^. Currently, two vaccines MVA-BN and ACAM2000 are being administered for mpox^6^. At present, the country has no vaccination facility but WHO has been requested to provide the vaccine for healthcare workers. Adequate arrangements must be made to arrange vaccines for high-risk populations, including individuals residing in flood relief camps and slums, via international collaboration, and to overcome vaccine hesitancy among the local population. A ring approach for such individuals is found to be beneficial in this regard^18^. It is equally crucial to enlighten the public to adopt preventive measures and report to the nearest clinic in case of similar symptoms. In case of an outbreak, strict standard operating procedures (SOPs) need to be implemented along with making the healthcare facility more accessible to the masses. To protect the already trembling system from this potential additional burden, collaborative efforts between researchers, fellow healthcare professionals, and policymakers are mandatory.

## Ethical approval

Ethics approval was not required for this commentary article.

## Consent

Informed consent was not required for this commentary article.

## Sources of funding

The author(s) received no financial support for the research, authorship, and/or publication of this article.

## Author contribution

S.H.A.: conceptualization; S.H.A., M.S., H.M., S.A., and M.A.H.: literature search and manuscript preparation; M.A.H.: supervision, conceptualization, and idea. All authors read and approved the final manuscript.

## Conflicts of interest disclosure

The authors have no conflicts of interest to declare.

## Research registration unique identifying number (UIN)

Not applicable.

## Guarantor

Md. Al Hasibuzzaman, ORCID: 0000-0003-0548-4836.

## Data availability statement

Not applicable.

## Provenance and peer review

Not commissioned, externally peer-reviewed.
